# Explaining the Links Between School Administrator Leadership, Job Satisfaction, and Participatory School Climate: A Machine Learning-Enhanced Multilevel Analysis of TALIS 2024 School Administrator Data

**DOI:** 10.3390/bs16071062

**Published:** 2026-06-26

**Authors:** Dönüş Şengür

**Affiliations:** Educational Science Department, Education Faculty, Firat University, Elazig 23200, Türkiye; dsengur@firat.edu.tr

**Keywords:** participatory school climate, school administrator leadership, job satisfaction, explainable machine learning, multilevel mediation analysis, TALIS 2024

## Abstract

A participatory school climate refers to the involvement of school administrators, teachers, and other school members in decision-making processes, their sharing of responsibility, and their collaborative work for school improvement. Since this climate can be related to individual, organizational, and contextual factors such as leadership, job satisfaction, diversity beliefs, workload, well-being, and national context, identifying the key variables that support a participatory school environment is important. This study used TALIS 2024 school administrator data to identify the main predictors of participatory school climate and examined the mediating role of school administrator job satisfaction in the relationship between school administrator leadership, used here in line with school principal leadership, and participatory school climate. The research is based on a two-stage analytical framework. In the first stage, explanatory machine learning analysis was conducted by comparing Elastic Net, Random Forest, and XGBoost models; the relative significance levels of the variables were evaluated using permutation importance and SHAP methods. In the second stage, mediation analysis was performed using multi-level linear mixed models, considering clustering at the national level; the indirect association was evaluated using bootstrap confidence intervals. The analyses were conducted using data from 16,335 school administrators. The findings showed that the highest prediction performance was produced by the XGBoost model and that model performance improved with the inclusion of the country variable. Explainability analyses indicated that school administrator leadership was the strongest predictor of participatory school climate, followed by job satisfaction and diversity beliefs. Multilevel models suggested that the association between school administrator leadership and participatory school climate was consistent, with an indirect pathway through school administrator job satisfaction; bootstrap findings also supported the statistical stability of this indirect association. These findings suggest that a participatory school climate is associated not only with individual perceptions but also with multifaceted conditions such as leadership, job satisfaction, inclusivity, and country context. By combining explanatory machine learning with multilevel statistical modeling, this study identifies variables associated with participatory school climate and examines an indirect association among leadership, job satisfaction, and participatory climate. Because TALIS survey weights and the full complex sampling design were not incorporated, the findings should be interpreted as associations observed in the pooled analytical sample rather than as population-representative estimates for participating education systems.

## 1. Introduction

Schools today are not merely institutions where instruction takes place. They are also organizational structures that manage change, organize collaboration, and support shared responsibility. Therefore, a participatory school climate holds an important place in the educational management literature. A participative school climate implies the active participation of school administrators, teachers, and other school workers in decision-making, collective responsibility, and joint efforts for the growth of schools. Although students are considered an important part of the larger school community influenced by such a school climate, they are not considered participants in measuring this construct. Studies on school climate and school culture show that supportive and trust-based school environments are associated with organizational effectiveness (MacNeil et al., 2009; Bryk & Schneider, 2002; Moolenaar, 2012). TALIS findings further indicate that school life quality, collaboration, and leadership practices are closely interconnected across diverse educational systems (OECD, 2020).

In this study, the term “school administrator” is used in accordance with the terminology used in the TALIS 2024 school administrator dataset and the composite indicators developed by the OECD. However, the structure examined here is not, in the narrow sense, bureaucratic school management, routine administrative procedures, or traditional school management. The focus of the study is on leadership practices exhibited by school administrators. These practices encompass leadership-oriented behaviors such as supporting common goals, strengthening cooperation, encouraging participation, and contributing to school development. Therefore, the phrase “school administrator leadership” is used in this study to refer to leadership practices reported by school administrators within the TALIS framework. This distinction is important because in the contemporary educational literature, the roles of school principal and school administrator are increasingly addressed from an educational leadership perspective rather than a traditional understanding of school management.

In this study, a participatory school climate refers to the involvement of school leaders, teachers, and other employees in the processes of decision-making, sharing responsibilities, and collaborating for the purpose of improving the school. Even though it is acknowledged that students play an important role in the overall functioning of the school community under the influence of this type of climate, they do not become participants in assessing the construct directly. In their recent study based on TALIS data, Jiang and Liu (2024) have found that school climate is connected with school context, principal’s features, and principal’s leadership practices, thus emphasizing the topicality of the issue in terms of the modern educational leadership literature. Previous studies related to school climate and culture have shown that the creation of supportive climates plays an important role (MacNeil et al., 2009; Bryk & Schneider, 2002; Moolenaar, 2012).

In this context, school administrator leadership can be considered one of the key determinants of participatory school climate. Studies in the field of educational leadership show that school administrators are not only individuals who carry out administrative tasks; they are also actors who create instructional orientation, strengthen common goals, and support collaboration (Hallinger, 2005). Similarly, transformational and distributed leadership approaches emphasize that school development progresses through interactive and shared processes, not individual ones (Leithwood & Jantzi, 2005; Spillane, 2006). Recent research continues to support the importance of school leadership for collaboration, innovation, and organizational quality across educational systems (Veletić et al., 2023; Hsieh et al., 2024; UNESCO, 2024). Current TALIS-based findings also suggest that school leadership is related to innovation in school practices and to stronger collaboration among teachers, school administrators, and other school staff (Hsieh et al., 2024). Job satisfaction also stands out as an important variable in the relationship between leadership and school climate. The job demands-resources approach suggests that employee well-being and job satisfaction are closely related to conditions such as workload, support, autonomy, and collaboration (Bakker & Demerouti, 2017). TALIS-based research in education also shows that a safe school climate and a collaborative and participatory school culture are important sources of job satisfaction, while stress and professional barriers weaken job satisfaction (Admiraal & Røberg, 2023). OECD reports also detail the links between working conditions, stress, job satisfaction, and school climate for teachers and school leaders (OECD, 2019, 2020). Therefore, it is thought that the relationship between school leader leadership and participatory school climate can also operate through school leader job satisfaction.

Several existing studies provide a robust basis for an in-depth analysis of school climate, educational leadership, and job satisfaction. First, a series of studies on school climate have demonstrated that the school environments marked by a culture of support, trust, and collaboration have positive connections to organizational efficiency, teachers’ voice and sense of ownership, as well as to school success (Bryk & Schneider, 2002; MacNeil et al., 2009; Moolenaar, 2012). In addition, studies of instructional, transformational, and distributed leadership have emphasized the crucial importance of school leaders in goal setting, professional cooperation among teachers, and creating the necessary organizational climate (Hallinger, 2005; Leithwood & Jantzi, 2005; Spillane, 2006). Finally, several studies focused on job satisfaction and the JD-R framework have shown a significant correlation between working conditions, support, autonomy, workload, and collaboration and professional satisfaction (Bakker & Demerouti, 2017; Admiraal & Røberg, 2023).

This research paper advances previous knowledge in several important respects. Firstly, it compares the relative significance of leadership, job satisfaction, workload, diversity attitudes, and well-being in promoting participatory school climate rather than studying them independently from each other. Secondly, it uses explainable machine learning and multilevel mediation analysis to identify robust predictors and then to explore a theoretically relevant mechanism by which school administrator leadership and job satisfaction affect participatory school climate. Lastly, it takes into account country context as another important factor in shaping school climate. The consideration of the country context is vital since participatory school climate can emerge based not only on individual and organizational factors but also on the larger educational environment and administrative practices, policies, and norms. It is possible to do so thanks to the international nature of the dataset like TALIS, while in previous studies, this aspect was considered either implicitly or separately from each other. Hence, this study builds upon the previous literature on school climate, school administrator leadership, and job satisfaction by looking at how individual, organizational, and contextual factors affect participatory school climate among TALIS 2024 school administrators.

A participatory school climate refers to an organizational structure where school members participate in decision-making processes, share responsibility, and view school development as a collaborative effort (Somech, 2010). This structure reflects not only the quality of interpersonal relationships but also the school’s capacity for collective action. Schools with a strong participatory climate are reported to have more visible teacher voices, more openly shared goals, and more sustainable school development efforts (Woolner et al., 2007). Therefore, school climate is considered one of the fundamental concepts in the literature on school development and organizational learning (Hoy et al., 2002; Tschannen-Moran, 2014; Westheimer, 2008). The participative school environment has much to do with the social capital and trust between school administrators, teachers, and all members of the teaching faculty at the schools. In this context, social capital refers to those interpersonal relationships that form within an institution, including the common norms, trust, professional networks, and collaborative intentions of the participants in such networks. Trust enables cooperation, which allows for problem solving and responsibility together. Thus, participative school environment can be defined as more than a preferred management style; rather, it is a pattern of social relations. In particular, the quality of relationships among teachers, shared norms, and the belief in collective effectiveness are shown among the elements that strengthen the participatory structure of the school (Bryk & Schneider, 2002; Goddard et al., 2004; Moolenaar et al., 2010).

In this study, a participatory school climate is not directly equated with innovation. However, it is thought that characteristics such as participation, collaboration, and shared responsibility are fundamental organizational conditions that support the school’s capacity for change. Indeed, a recent study conducted with TALIS 2018 school administrator data showed that collaborative school culture, shared responsibility, and participatory decision-making processes stand out among the strongest determinants of innovation at the school level (İncekara et al., 2026). This finding suggests that a participatory school climate offers a central context for the school’s capacity for development and renewal.

### Organizational and Individual Factors Influencing the Participatory School Climate

One of the main organizational variables affecting a participatory school climate is school administrator leadership (Griffith, 1999). Leadership is a process that determines the direction of the school, makes shared goals visible, and encourages the participation of school members. The instructional leadership approach, in particular, emphasizes that the school administrator is not limited to administrative tasks; they also undertake responsibilities such as supporting instruction, promoting professional development, and creating a positive school climate (Hallinger, 2005; Horng & Loeb, 2010). Similarly, the distributed leadership approach argues that school development is based not on the actions of a single administrator but on interaction networks and shared leadership processes within the school (Spillane, 2006). Therefore, school administrator leadership is expected to have a direct contribution on a participatory school climate.

Job satisfaction can be seen as an important individual mechanism in the relationship between leadership and school climate. Job satisfaction reflects the administrator’s level of commitment to their school, the meaning they derive from their work, and how they experience the school environment. In educational organizations, job satisfaction is closely related to supportive relationships, sense of professional competence, workload, and working conditions. The job demands-resources approach also offers a suitable framework for explaining this relationship. According to this approach, elements such as support, autonomy, and collaboration function as job resources, while excessive workload and role pressures can weaken job satisfaction (Bakker & Demerouti, 2017). In contexts where school administrators have high job satisfaction, the likelihood of the school having a more positive, more participatory, and more supportive structure may also increase (F. Wang et al., 2018; A. Maxwell & Riley, 2017).

Another factor that can affect a participatory school climate is school administrators’ workload (Werang, 2018). A heavy workload can reduce the time and energy that the administrator can dedicate to relational leadership practices. This can limit participation in decision-making processes and lead to a more bureaucratic operation in the school. In educational leadership literature, workload, burnout, and emotional labor are cited as significant variables shaping the work experience of school administrators (A. Maxwell & Riley, 2017; Marsh et al., 2023). Therefore, workload can be expected to be negatively correlated with a participatory school climate.

Beliefs about diversity are also important for a participatory school climate (Parris et al., 2018). A school that is open to differences, views diverse perspectives as resources rather than threats, and produces an inclusive culture can support a more participatory school structure. Culturally sensitive school leadership literature draws attention to the role school administrators play in inclusivity, justice, and representation (Khalifa et al., 2016). In this context, it is stated that open and inclusive beliefs about differences may be associated with more democratic and participatory school environments (Khalifa et al., 2016; Khalifa, 2020). Well-being is a multidimensional concept that refers to an individual’s positive evaluation of their life from an emotional, psychological, and social perspective (Kiefer, 2008). Not merely the absence of negative experiences or stress, well-being is also related to an individual feeling adequate, valuable, and meaningful.

In this context, well-being encompasses elements such as experiencing positive emotions, high life satisfaction, supportive interpersonal relationships, and the ability to effectively cope with challenges. In particular, high levels of well-being in educational settings are considered a significant factor in strengthening individuals’ motivation, sense of belonging, and overall functioning (Prince & Hadwin, 2013).

Finally, the national context can influence all these relationships. International datasets such as TALIS show that variables such as school leadership and school climate are shaped not only at the individual or school level but also at the systemic level. National education policies, administrative culture, accountability structures, and professional norms can be associated with the leadership practices of school administrators and the school climate. Therefore, country context should be considered in models describing participatory school climate (OECD, 2019, 2020).

## 2. Research Questions

The main objective of this study is to determine which variables are most strongly associated with participatory school climate in TALIS 2024 school administrator data. To this end, the study is based on a two-stage analytical framework. In the first stage, the main predictors of participatory school climate are revealed using an explainable machine learning approach. In the second stage, the mediating role of school administrator job satisfaction in the relationship between school administrator leadership practices and participatory school climate is tested using multi-level analysis. This approach allows for determining the relative importance of variables in complex data structures, on the one hand, and explaining how theoretically meaningful relationships operate, on the other. This is the main reason why explainable artificial intelligence and interpretable modeling approaches are increasingly used in educational research: these approaches not only generate predictions but also provide interpretable findings for educational decision-making processes. Within this framework, the study sought answers to the following research questions:

RQ1. What are the strongest predictors of participatory school climate in TALIS 2024 school administrator data, as identified through explainable machine learning models?

RQ2. Does school administrator job satisfaction mediate the relationship between school administrator leadership and participatory school climate when the multilevel country context is taken into account?

Based on the research questions, the findings may inform educational leadership research and school improvement efforts by clarifying the roles of leadership practices, job satisfaction, and country context in predicting and explaining participatory school climate.

As part of defining the research questions, this study will focus on those variables within the 2024 TALIS survey on the school administrator dataset that align with the concept of participatory school climate. More specifically, such variables include school administrator leadership, job satisfaction, workload, diversity beliefs, well-being, country context, and sample population. While other variables, such as tenure, salary, training, race/ethnicity, and school funds, may be related to school climate and job satisfaction, they could not be used in the analysis of this study due to the lack of availability as compared to the TALIS composite indicators. This means that the results provided herein are expected to be estimates based on the defined predictors rather than being fully adjusted estimates of causal effects. Based on the literature review and theoretical background, the following hypotheses can be advanced:

**H1.**  *There is a difference in the relative importance of school administrator leadership, job satisfaction, diversity beliefs, well-being, workload, and country context as predictors of participatory school climate, with school administrator leadership as one of the strongest predictors.*

**H2.**  *School administrator job satisfaction will mediate the relationship between school administrator leadership and participatory school climate, while adjusting for workload, diversity beliefs, and country-level clustering.*

### Research Model

The research model was developed in response to the gap in the body of research on educational leadership and school climate. Although previous studies have examined the correlations between school leadership, job satisfaction, and school climate, such relationships were typically analyzed through traditional one-stage modeling. At the same time, there have been not too many attempts to use explainable machine learning in combination with multi-level mediation analysis for determining the significance of various predictors and uncovering the process that connects leadership with participatory school climate. For this reason, the two-stage model was selected for providing both types of results based on TALIS 2024 school administrator data.

This study’s research model is based on explaining the relationships between school administrator leadership, school administrator job satisfaction, and participatory school climate. In the model, school administrator leadership is considered the independent variable, school administrator job satisfaction the mediating variable, and participatory school climate the dependent variable. In addition, workload and diversity beliefs are included in the model as control variables. To consider the effect of contextual differences between countries, the analyses were conducted within a multi-level structure. Thus, not only the individual characteristics of school administrators but also the national context in which they operate have been made part of the model. This approach is important in terms of considering the multi-layered nature of the relationship between leadership and school climate. The first dimension of the research model aims to identify the main predictors of participatory school climate. For this purpose, an explainable machine learning approach was used. Explainable machine learning offers a significant opportunity for educational research because it can reveal nonlinear relationships in large-scale and multivariate data structures. However, this approach alone should not be considered sufficient because determining the order of importance of variables is not the same as explaining how these variables work together. Therefore, in the present study, machine learning findings are complemented by a multi-level mediation model in the second phase. Thus, the study answers not only the question “Which variable is more important?” but also “Through what mechanism does this relationship operate?”

The second dimension of the research model examines whether the association between leadership and participatory school climate is consistent with an indirect pathway through job satisfaction. The assumption is not that leadership causally produces participatory climate, but that leadership practices and job satisfaction may be systematically related to more participatory school conditions in the TALIS 2024 cross-sectional data. In other words, strong leadership practices may not only directly affect organizational processes; they may also increase the administrator’s positive evaluations of their work and psychosocial resources. This can contribute to the creation of a more participatory and supportive climate in the school. Therefore, the inclusion of workload and diversity beliefs as control variables in the model is also important. Workload can be a pressure factor that may limit the administrator’s participatory and relational leadership practices; diversity beliefs, on the other hand, can support a more inclusive and participatory school environment.

## 3. Material and Methods

### 3.1. Research Design

This study is designed as a secondary data analysis within a quantitative research approach. The analytical framework consists of two stages. In the first stage, an explainable machine learning approach was used to identify the main predictors of participatory school climate. In the second stage, the mediating role of school administrator job satisfaction in the relationship between school administrator leadership and participatory school climate was examined using multi-level modeling. This two-stage design allows for both revealing the relative importance of variables in complex data structures and explaining how theoretically meaningful relationships operate (Lundberg & Lee, 2017; Türkmen, 2025).

### 3.2. Data Source and Sample

This study utilizes TALIS 2024 school administrator data from the fourth cycle of the Teaching and Learning International Survey conducted by the OECD. The TALIS 2024 survey involves a variety of education systems, which participate in an international survey of teachers and school leaders organized by the OECD. The dataset used for analysis includes the education systems of Albania, Austria, Azerbaijan, Bahrain, Brazil, Bulgaria, Chile, Colombia, Costa Rica, Croatia, Cyprus, Czechia, Denmark, Estonia, Finland, France, Belgium, Hungary, Iceland, Israel, Italy, Japan, Kazakhstan, Korea, Kosovo, Latvia, Lithuania, Malta, Montenegro, Morocco, the Netherlands, New Zealand, North Macedonia, Norway, Poland, Portugal, Romania, Saudi Arabia, Serbia, Singapore, the Slovak Republic, Slovenia, South Africa, Spain, Sweden, Türkiye, the United Arab Emirates, the United States, Uzbekistan, and Vietnam. Despite differences between the included education systems concerning culture, governance, and policies, all of these systems are involved in conducting an international standardized survey aiming at obtaining equivalent data from teachers and school leaders. Thus, the results of the survey should be applied only to those education systems participating in TALIS 2024.

TALIS 2024 is a large-scale study that comparatively examines the working conditions, professional development, leadership practices, and perceptions of school life of teachers and school administrators on an international scale (OECD, 2025a, 2025b). In the present research, analyses were conducted using a combined data file of school administrators. The analytical dataset contains 16,335 observations and 8 variables based on the variables used in the study. This structure, unlike previous narrowed subsamples, is based on a broader principal data frame that brings together three separate sample populations. When the IDPOP variable is examined, it is seen that 3684 observations belong to the first population, 11,272 to the second, and 1379 to the third. In the machine learning phase, the preservation of valid observations for the dependent variable was prioritized. Therefore, all observations with valid values in the participant school climate variable were included in the analysis process. In multilevel mediation analysis, observations with complete data in terms of independent variable, mediating variable, dependent variable, and control variable were used. The country variable was used both to provide contextual information in the machine learning phase and to define the clustering structure in multilevel models. In this respect, the data structure offers a multi-layered structure suitable for examining not only individual differences but also contextual differences between countries. Indeed, model comparisons have shown that the prediction performance increases significantly when country information is included in the model. This supports the fact that the TALIS data structure is suitable for context-sensitive multilevel analyses (OECD, 2025b, 2025c).

Since the TALIS 2024 data is a large-scale international study based on a complex sampling structure, the possible association of the sample design on the analytical results was also taken into consideration. However, since the primary aim of the present study is not to generate descriptive estimates based on country representation but rather to explain the patterns among variables related to the participatory school climate, and to examine the relative importance of these relationships, the analyses were conducted using a combined data file. Country-level clustering effects were included in the model through multi-level models; thus, structures where observations are not independent were partially controlled. However, since not all components of the sample weights and complex sampling design were directly reflected in the model, it would be more appropriate to interpret the findings primarily in the context of relational patterns between variables. Therefore, the research results should be evaluated not as weighted estimates directly generalized to the entire TALIS universe but as comparative and explanatory findings emerging within an international data structure.

### 3.3. Variables

In this study, participatory school climate (T4POPPART) was used as the dependent variable (Appendix A). The independent variable was defined as school administrator leadership (T4PTLEAD), and the mediating variable as school administrator job satisfaction (T4PJOBSAT). School administrator workload (T4PWLOAD); diversity beliefs (T4PDIVBF); and, in the machine learning phase, school administrator well-being (T4PWELS) were included in the model as control variables. In addition, the country variable (CNTRY) was used to provide contextual information and define the multi-level structure, and the sample population variable (IDPOP) was added to the model to control for different population structures in the combined data file. Thus, in the analyses, not only individual and organizational indicators but also differences regarding the sample structure of the dataset were taken into account (OECD, 2025b, 2025c).

The selection of variables was made to focus on indicators that are theoretically related to participatory school climate and interpretable in the field of educational administration. School administrator leadership, job satisfaction, workload, and well-being are considered indicators directly related to the organizational functioning and leadership processes of the school. Beliefs about diversity are seen as important for understanding inclusive and participatory school environments. This approach is consistent with the structure of the TALIS database, which allows for the joint evaluation of school, leadership, and working conditions (OECD, 2025a, 2025b, 2025c). The main variables used in the research are based on composite indicators scaled by the OECD within the framework of TALIS 2024. These scales were created by considering the psychometric properties of the responses given to the relevant item groups and have been standardized for use in international comparisons. Therefore, in the present study, the variables T4POPPART, T4PTLEAD, T4PJOBSAT, T4PWLOAD, T4PDIVBF and T4PWELS were interpreted not as directly observed individual items but as scale scores representing theoretically defined latent constructs. In this study, the measurement structure of these scales reported in the OECD technical documentation was used as the basis; thus, conceptual and measurement-based consistency of the indicators used in the analyses was ensured.

The key variables used in this study are composite indices that have been developed using the OECD-scaled TALIS 2024 data and not item-based constructs created by the researchers. As such, the values of the Cronbach’s alphas cannot be computed anew using the analysis data file because the computation of the Cronbach’s alpha requires the use of item-based variables, which were not included in the analysis data file. For this reason, the current study had to rely on the procedures described for the development of scales in the TALIS technical documentation.

### 3.4. Data Preparation Process

Firstly, any missing or invalid responses in the TALIS data were cleaned using Python 3.11.15 software, and all those coded values were redefined as missing data. All variables used in this analysis were first converted into numerical variables. Missing data patterns were inspected before estimation of the regression models. In the original data file, missing data patterns varied, from 2.64% in school administrator leadership to 3.75% in school administrator job satisfaction. Records with missing data in the dependent variable were removed from the machine learning analysis sample. After filtering the dependent variable data, there were 16,335 records included in the machine learning analysis. The predictor variables contained very little missing data: 0.77% in job satisfaction, 0.64% in workload, 0.62% in well-being, 0.55% in diversity belief, and 0.18% in school administrator leadership. The complete-case missing data pattern covered 98.86% of the machine learning analysis sample. With regard to the residual missing data which occurred only in predictors, median imputation was used.

Median imputation technique was used only in the machine learning process. The analysis of multilevel mediational paths was based on cases without any missing data concerning the variables involved in the mediational model. Due to the low levels of missing data following the elimination of data having missing data on the outcome variable from 0.18% to 0.77%, multiple imputation techniques were not used in this research.

Country and sample population variables were converted into appropriate representation formats for use in the analyses. Due to the different ranges of the scales, standardization was applied to ensure comparability, especially in linear and regularized models. In the multilevel mediation analysis, centralization was performed according to the country average to separate within-country and between-country effects. In this context, both the deviations within the country and the average values at the country level were calculated for the variables of leadership, job satisfaction, workload, and diversity beliefs. Thus, on the one hand, the positions of school administrators according to the average level in their own countries and, on the other hand, contextual differences between countries could be included in the model. This approach contributes to a more accurate separation of individual-level effects and contextual effects in international comparative data structures (OECD, 2019, 2025a).

### 3.5. Machine Learning Methods

Supervised machine learning is a modeling strategy in which an algorithm learns the relationship between predictor variables and a known outcome variable. In this study, the outcome variable is participatory school climate, while the chosen TALIS measures are the predictors. The two goals of using the method are to make predictions based on the dependent variable and identify which predictors contribute more to prediction accuracy.

The Elastic Net model is a regularized regression model which combines ridge regression and lasso regression. While ridge regression minimizes overfitting through shrinking the parameters, lasso regression sets some parameters at zero, thus aiding in selecting the variables. The Elastic Net technique was included in this research for purposes of comparing it with the tree machine learning techniques.

In the first phase of this study, a supervised machine learning approach was used to identify the variables that most strongly predict school climate. For this purpose, three different regression algorithms were compared: Elastic Net, Random Forest, and XGBoost (De Mol et al., 2009; Rigatti, 2017; Chen et al., 2015). This model family was deliberately chosen. Elastic Net is a method based on linear relationships but limits overfitting through regularization. Random Forest, with its ensemble learning logic based on multiple decision trees, can capture nonlinear patterns and interactions between variables. XGBoost, on the other hand, is a powerful boosting algorithm that works with incremental tree structures and offers high predictive power in complex data structures. Thus, both linear and nonlinear modeling approaches were compared on the same dataset. In machine learning analyses, school climate was used as the dependent variable. Predictive variables included school administrator leadership, job satisfaction, well-being, workload, diversity beliefs, and variables representing the country and sample population. Categorical variables were appropriately converted into dummy variables, and missing data were handled using a central tendency-based approach in the modeling process.

In the Elastic Net model, variables were scaled to a common scale. In tree-based models, direct modeling was performed after completing missing data. The dataset was divided into training and test sets with an 80–20% ratio, hyperparameter selection was performed only on the training data, and the test data was saved for independent final evaluation. Five-fold cross-validation was used in the model tuning process. The best model was selected based on the average coefficient of determination obtained in the cross-validation process. The final model performance was evaluated in the independent test set using the coefficient of determination, mean absolute error, and root mean square error. The adjustment ranges used in model comparison were predefined. In the Elastic Net model, values of 0.01, 0.10, and 1.00 were tested for the penalty level representing the strength of regularization and values of 0.20, 0.50, and 0.80 were tested for the ratio reflecting the balance of the L1 and L2 regularization components.

In the Random Forest model, values of 200 and 400 were evaluated for the number of trees; 5, 10, and unlimited structure for the maximum tree depth; and 2 and 5 for the node splitting threshold. In the XGBoost model, values of 200 and 400 for the number of trees; 3, 5, and 7 for the maximum depth; 0.03 and 0.10 for the learning rate; and 0.80 and 1.00 for the downsampling rate were tested. Thus, a limited but theoretically meaningful calibration space was explored for each model; an attempt was made to strike a balance between model complexity and generalizability. Finally, each algorithm was retrained with the calibration combination that provided the highest average performance in the cross-validation process and evaluated on the test set.

### 3.6. Explainability Analysis

After comparing the machine learning models in the first analytic phase of the research, the interpretability of the selected models was examined. This explainability step remained part of the machine learning phase and should be distinguished from the second analytic phase of the study, in which multilevel mediation analysis was conducted. In educational research and social sciences, it is accepted that predictive power alone is insufficient; it is also necessary to show which variables the model relies on, to what extent, and in what direction. Accordingly, two complementary explainability methods were adopted in this study, namely permutation importance (Altmann et al., 2010) and SHAP (Gilpin et al., 2018). The first approach is based on observing the deterioration in model performance by randomly rearranging the value of each variable. The greater the decrease in performance, the more important the relevant variable is considered to be for the model. This method is particularly useful in showing the relative importance of variables at the global level. In this study, permutation importance results were calculated for models both with and without country variables. The results showed that in both cases, school administrator leadership was the strongest predictor, followed by job satisfaction. When country variables were included in the model, it was observed that some country dummy variables also made a high contribution. This indicates that the participatory school climate is related not only to individual leadership and well-being variables but also to broader contextual conditions. Thus, permutation importance analysis revealed the overall ranking of variable importance. The second explainability approach, SHAP, allowed for a more detailed interpretation of model predictions. SHAP analysis calculates the contribution of each variable to the model prediction at the level of individual observations and also shows the direction of this contribution. Thus, it can be seen not only which variable is important but also whether high or low values of the relevant variable affect the model prediction in an increasing or decreasing direction. In this study, SHAP visualizations were produced through the Random Forest model due to technical compatibility. However, the obtained ranking of importance largely coincided with the permutation importance findings. SHAP summary graphs showed that school administrator leadership, job satisfaction, and diversity beliefs were particularly prominent variables in model predictions and that some country variables carried directional contextual effects. In this respect, SHAP analysis made visible not only which variables the participant school climate is related to but also how these relationships are reflected in the model prediction. When these two components of explainability analysis are evaluated together, the machine learning phase not only determined the “best prediction model”; it also showed which individual and contextual variables were more strongly associated with the participatory school climate. These findings provided the empirical basis for the multilevel mediation analysis conducted in the second phase of the study. In other words, the machine learning results revealed that leadership and job satisfaction were central variables; the multilevel mediation analysis then tested how the relationship between these two variables affected the participatory school climate. Thus, explanatory machine learning analysis and multilevel mediation analysis were used as two complementary phases (Lundberg & Lee, 2017; Türkmen, 2025).

### 3.7. Multilevel Mediation Analysis

In the second phase of the research, the mediating role of school administrator job satisfaction in the relationship between school administrator leadership and participatory school climate was tested using multi-level linear mixed models. In this phase, the country was defined as a random intercept level. In the first model, school administrator job satisfaction was considered as the mediating variable, and the effects of leadership and control variables on job satisfaction were tested. In the second model, participatory school climate was considered as the dependent variable; the effects of leadership, job satisfaction, and control variables on this variable were examined together. The indirect effect was calculated through the product of paths a and b. The reason for choosing a multi-level modeling approach was that the observations showed a hierarchical structure clustered according to countries. Since the analysis involves an international comparative database and aims to control for differences between education systems, the country was used as the clustering unit. It should, however, be noted that clustering at the country level does not reflect all variations that exist at the level of context. For example, principals may experience different things based on the characteristics of the local environment, such as the rural/urban nature of the environment, the particular region within a country where they live, availability of school resources, or district characteristics. Therefore, the country level was defined as a random intercept in the models, thus aiming to separate both within-country and between-country variance. In interpreting the model results, both fixed effect coefficients and random effect components were considered; the presence of between-country variance showed that participatory school climate also carries a contextual pattern. In the assessment of the multilevel models developed, the focus was on fixed effect estimates, standard error, significance of variables, convergence of the mixed models, as well as variance components, which included the random intercept at the country level and residual variance. The use of these variance components helped determine if there is any between-country variation in the dependent variable after controlling for individual level predictors. Considering that the mediation models were based on theories rather than comparison of competing models, information criteria like AIC and BIC were not applied in model selection. This separated the direct contribution of leadership on participatory school climate from its indirect impact operating through job satisfaction. Separating intra-country and country-level effects is important, particularly in multi-country datasets such as TALIS, to reduce misinterpretation of contextual effects (Hsieh et al., 2024; OECD, 2025a).

### 3.8. Validity and Robustness Checks

The indirect association was evaluated primarily using bootstrap confidence intervals and the size of the indirect coefficient. This approach allowed the mediation finding to be evaluated not only based on point estimation but also within a robustness framework based on resampling. In particular, the bootstrap method was used to examine the extent to which the indirect effect could be stably reproduced across different sample repetitions. In the multilevel mediation analysis, variables centered relative to the country average were used to separate within-country and country-level effects. This process allowed the school administrator’s position relative to the average level in their own country to be separated from the average differences between countries. Especially in multi-country datasets such as TALIS, this approach reduces the mixing of individual-level relationships with contextual-level patterns and contributes to a more accurate interpretation of the results (Hsieh et al., 2024; OECD, 2025a). In addition, maintaining the multilevel structure was considered important to reduce standard error biases that may occur due to clustering of observations by country and to strengthen the contextual consistency of the findings. Thus, the mediation findings were interpreted by testing them through both a modeling approach and validation steps based on resampling.

## 4. Results

This section presents the findings obtained from the explainable machine learning and multi-level mediation analyses conducted within the scope of the research. In the first stage, the performance of the models predicting the participatory school climate was compared, and then, the relative importance levels of the independent variables were evaluated within the framework of explainability analyses. In the second stage, the mediating role of school administrator job satisfaction in the relationship between school administrator leadership and participatory school climate was examined through multi-level models. The findings are reported in a systematic and integrated structure in line with the objectives and two-stage analytical framework of the study.

### 4.1. Comparison of Machine Learning Models

Table 1 shows the performance of machine learning models designed to predict the school climate on test data. Coefficient of determination (R^2^), mean absolute error (MAE), and root mean square error (RMSE) measures were used to evaluate the models. The R^2^ value reveals the extent to which the model explains the variance in the dependent variable, while the MAE and RMSE values provide information about the error level in the model predictions (Chicco et al., 2021). In this context, a high R^2^ value and low MAE and RMSE values indicate stronger model performance (Willmott & Matsuura, 2005).

Examining the findings in Table 1, it is seen that the highest performance is obtained by the XGBoost model in both data structures. In the analyses where the country variable was included in the model, the R^2^ value of the XGBoost model was determined to be 0.218, the MAE value 1.417, and the RMSE value 1.794. In the same data structure, it is understood that the ElasticNet model also exhibited a performance quite close to XGBoost, while the Random Forest model has a relatively lower explanatory power. This situation suggests that both linear and nonlinear patterns can be effective in explaining the school climate of the participants, but the strongest prediction performance is produced by the boosting-based approach. When the country variable is not included in the model, a significant decrease in the performance of all models is observed. In this condition, although the highest R^2^ value belongs to the XGBoost model, it is seen that the explained variance ratio decreased to 0.123. Similarly, the increase in MAE and RMSE values indicates that the model error level has increased. In particular, the difference between the country-inclusive and country-exclusive models reveals that participatory school climate is related not only to individual or organizational variables but also to the national context.

Overall, the findings in Table 1 point to three important conclusions. First, the XGBoost model showed the most successful prediction performance in both datasets. Second, the fact that the ElasticNet model produced results close to XGBoost in the country-inclusive analyses suggests that the relationships between variables also have a linear structure to a certain extent. Third, the improvement in performance metrics seen with the addition of the country variable to the model supports the idea that participatory school climate is a context-sensitive variable and that this structure needs to be addressed at a multi-level in international comparative data. In this respect, Table 1 provides an important starting point for understanding why the national context was considered in the later stages of the research and why the explainable machine learning findings were complemented by multi-level analyses.

**Figure 1:**
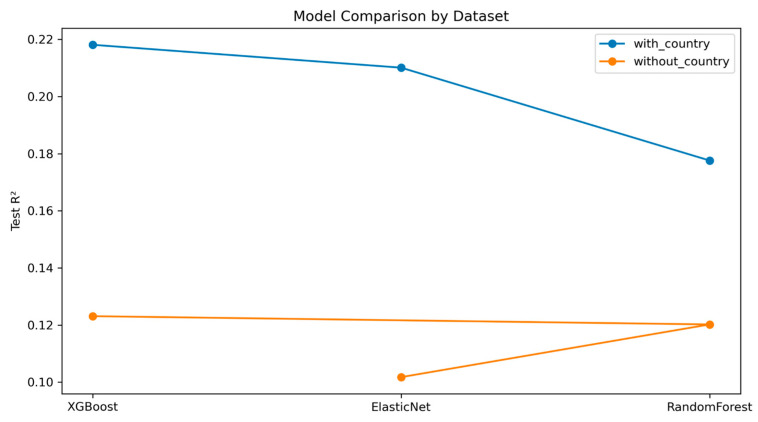
Comparison of test R^2^ values across machine learning models with and without the country variable. Numeric labels indicate the explained variance for each model, showing that model performance improved when country information was included.

Figure 1 shows a comparative analysis of the test R^2^ values produced by machine learning models designed to predict the participatory school climate, in two different data structures, one with and one without the country variable. As seen in the figure, all algorithms exhibited higher explanatory power when the country variable was included in the model. The highest performance was observed in the XGBoost model, followed by the ElasticNet and Random Forest models. When the country variable was excluded, a decrease occurred in the R^2^ values of all models. This finding demonstrates that the country context makes a significant contribution to explaining the participatory school climate and systematically strengthens model performance. Therefore, the figure visually supports the rationale for adopting a multi-level and context-sensitive analytical approach in the research.

**Figure 2:**
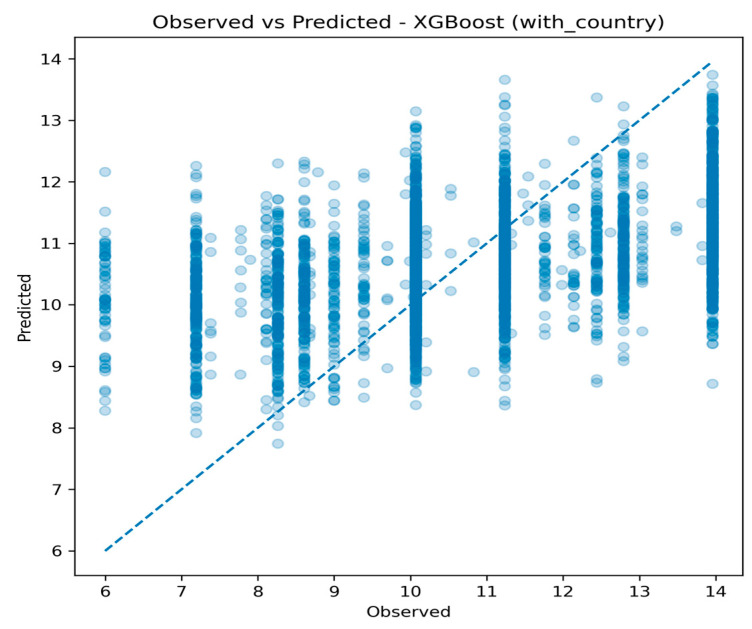
Observed and predicted values for the best model, including country.

Figure 2 illustrates the relationship between observed and predicted values in the best-case model, which includes the country variable. The clustering of points generally around the diagonal reference line indicates that the model was able to capture the scores related to the participant school climate to a certain extent. However, the fact that the points did not concentrate completely around the line shows that the prediction errors were not entirely eliminated. The continued scattering, especially in the medium and high observed values, suggests that the model may have underestimated or overestimated some observations. Nevertheless, the overall pattern reveals that with the addition of the country context to the model, the predictions approached the observed values more closely, and the explanatory power of the model was relatively strengthened.

**Figure 3:**
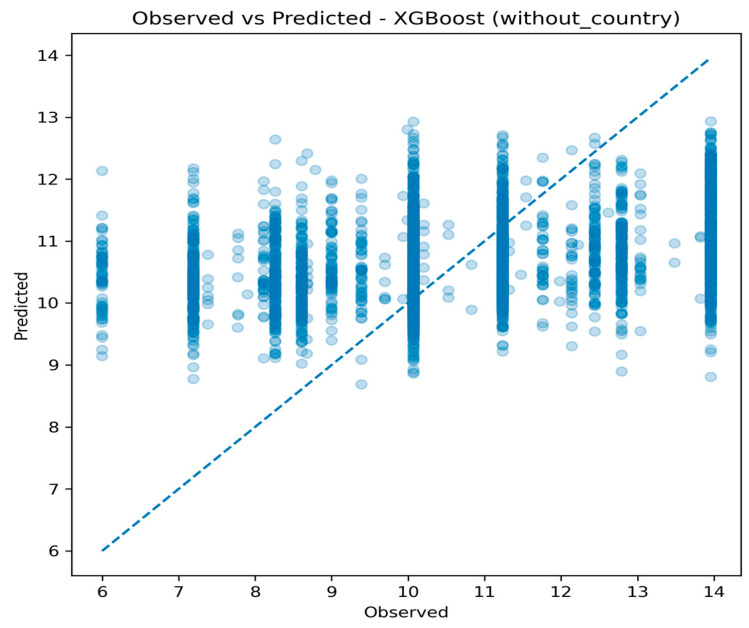
Observed and predicted values for the best model excluding country.

Figure 3 illustrates the relationship between observed and predicted values in the best-case model, where the country variable is not included. The clustering of points around the reference line reveals that the model’s ability to explain observed values is more limited compared to the model including the country. The concentration of predictions in specific bands suggests that the model’s capacity to discriminate variation in the participatory school climate is weakened. This visual pattern supports the idea that model performance decreases when the country context is excluded, demonstrating that the participatory school climate is a context-sensitive construct.

### 4.2. Variable Importance Ranking

Table 2 shows the variables that stand out in predicting the school climate of the participants, based on the permutation importance analysis, comparatively for two different model structures, one with and one without the country variable. Permutation importance is based on the decrease in model performance that occurs when the values of a variable are randomly mixed; accordingly, variables that cause a greater deterioration in performance are considered more important for the model. In this respect, Table 2 reveals not only which variables are effective but also the relative weight of these variables within the model.

Examining the findings in Table 2, it is seen that school administrator leadership (T4PTLEAD) is the strongest predictor in both model structures. The fact that this variable ranks first in both the country-inclusive and country-exclusive models indicates that leadership plays a central role in explaining the participatory school climate. Similarly, school administrator job satisfaction (T4PJOBSAT) also ranks highly in both models and stands out as an important variable following leadership. This finding suggests that the participatory school climate is closely related not only to managerial practices but also to the job satisfaction of the school administrator. In the structure where the country variable is included in the model, it is noteworthy that some country dummy variables, such as Denmark, Japan, and Korea, rank highly. This shows that the participatory school climate is shaped not only at the individual and organizational levels but also by the national context. The higher importance of Denmark, Japan, and Korea should be interpreted as a contextual pattern rather than as evidence of causal country effects. Denmark may reflect features often linked to Nordic education systems, such as trust, cooperation, and decentralized school administration, while Japan and Korea may reflect East Asian systems where school management, professional responsibility, and system-level coordination are strongly emphasized (OECD, 2020, 2025a, 2025c).

On the other hand, when the country variable is removed from the model, variables such as diversity beliefs (T4PDIVBF), well-being (T4PWELS), and workload (T4PWLOAD) become more visible. This pattern shows that when the country context is not controlled for, the model’s explanatory power is based more on intra-school relational and individual variables. Thus, Table 2 reveals that leadership and job satisfaction are key variables in explaining the school climate and that the country context also makes a significant contribution. These findings provide a strong empirical basis supporting the examination of the relationship between leadership and job satisfaction using multi-level mediation analysis in the next phase of the research.

**Figure 4:**
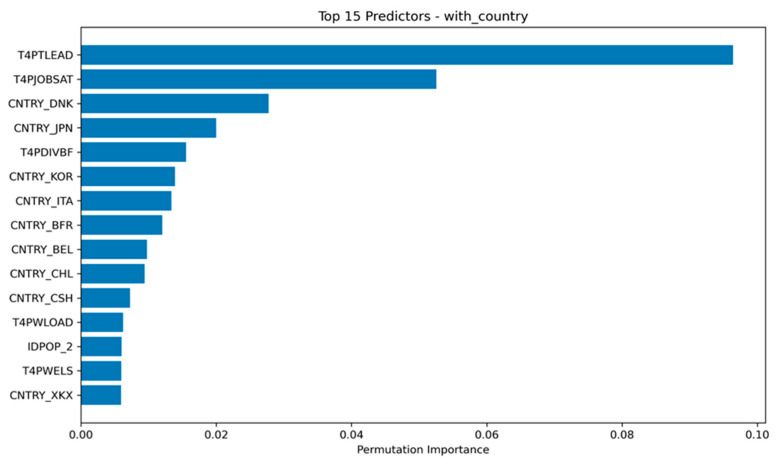
Top predictors in the country-inclusive model based on permutation importance. Numeric labels show the contribution of each predictor to model performance; higher values indicate greater importance for predicting participatory school climate. Note. T4PTLEAD = school administrator leadership; T4PJOBSAT = school administrator job satisfaction; T4PDIVBF = diversity beliefs; T4PWLOAD = school administrator workload; T4PWELS = school administrator well-being. CNTRY codes refer to country dummy variables.

Figure 4 shows the top 15 variables with the highest contribution to predicting the participatory school climate according to the permutation importance analysis when the country variable is included in the model. The graph visually demonstrates the relative contribution of each variable to the model performance; variables with higher permutation importance values show that they are more decisive for the model. In this respect, the figure visually complements the findings presented in Table 2 and allows for a clearer observation of the importance ranking of the variables. Examining Figure 4, it is seen that school administrator leadership (T4PTLEAD) is by far the strongest predictor. It is followed by school administrator job satisfaction (T4PJOBSAT) in second place. This finding shows that leadership practices and administrator job satisfaction play a central role in the formation of the participatory school climate. In addition, the high ranking of some country variables such as Denmark, Japan, Korea, Italy, and Belgium reveals that the country context has a significant explanatory power for the model. The inclusion of variables such as diversity beliefs (T4PDIVBF), workload (T4PWLOAD), and well-being (T4PWELS) suggests that participatory school climate is related not only to managerial leadership but also to individual and organizational characteristics. This finding supports previous studies in the area of educational leadership that show how school leadership is closely related to common objectives, cooperation among professionals, and school climate (Hallinger, 2005; Leithwood & Jantzi, 2005; Spillane, 2006). The pattern also corresponds with studies based on TALIS and OECD publications, showing how cooperation, working conditions, job satisfaction, and school climate influence one another within the experience of teachers and school leaders (Admiraal & Røberg, 2023; OECD, 2019, 2020). Therefore, the importance of leadership and job satisfaction in the current model support the findings from previous research that indicate how cooperative and supportive school climates are linked to school leadership and job satisfaction.

**Figure 5:**
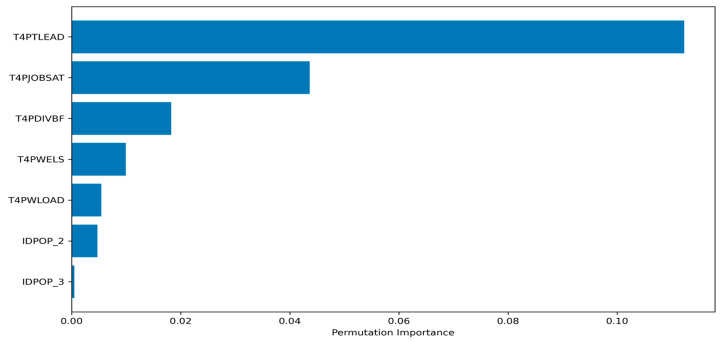
Top predictors in the country-excluded model based on permutation importance. Numeric labels indicate the relative contribution of each predictor when country information was not included in the model. Note. T4PTLEAD = school administrator leadership; T4PJOBSAT = school administrator job satisfaction; T4PDIVBF = diversity beliefs; T4PWELS = school administrator well-being; T4PWLOAD = school administrator workload; IDPOP = sample population.

Figure 5 shows the variables that stand out in predicting the participatory school climate according to the permutation importance analysis when the country variable is not included in the model. The graph visually presents the relative importance levels of the variables that contribute most to the model performance and reveals which variables become more prominent when the country context is excluded. In this respect, the figure contributes to comparison with the findings obtained in the country-included model and to understanding which structures the model operates through in the absence of contextual variables. When Figure 5 is examined, it is seen that school administrator leadership (T4PTLEAD) is by far the strongest predictor in the country-excluded model as well. School administrator job satisfaction (T4PJOBSAT) ranks second, followed by diversity beliefs (T4PDIVBF), well-being (T4PWELS), and workload (T4PWLOAD). This finding shows that when the country context is not included in the model, the participatory school climate is explained more through intra-school relational, individual, and organizational variables. The high ranking of diversity beliefs and well-being, in particular, indicates that a participatory school climate is fueled not only by managerial leadership but also by dimensions such as perceived inclusivity and psychosocial well-being. However, the exclusion of country variables, which are prominent in the country-specific model, suggests that the explanatory burden shifts directly to individual and school-level variables by removing contextual differences from the model. Overall, the model reveals that leadership and job satisfaction maintain their central importance in both model structures; however, the relative visibility of other organizational and individual variables increases when the country context is excluded.

### 4.3. SHAP Analysis

**Figure 6:**
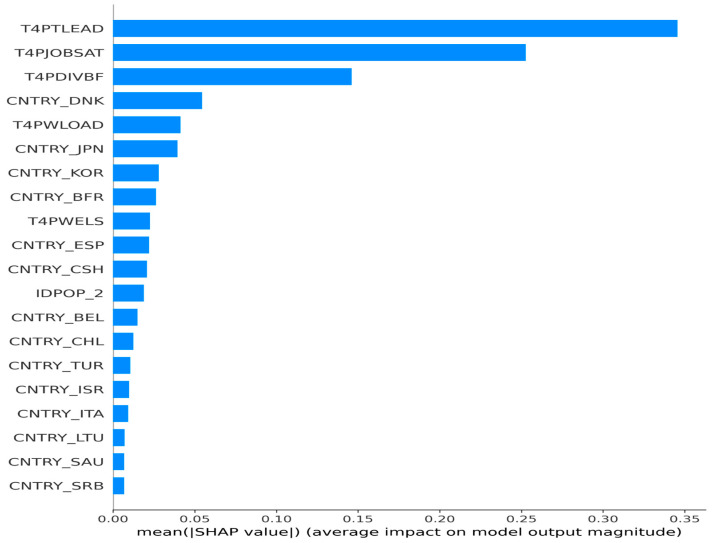
Mean absolute effect sizes of variables according to SHAP analysis. Note. T4PTLEAD = school administrator leadership; T4PJOBSAT = school administrator job satisfaction; T4PDIVBF = diversity beliefs; T4PWLOAD = school administrator workload; T4PWELS = school administrator well-being. CNTRY codes refer to country dummy variables.

**Figure 7:**
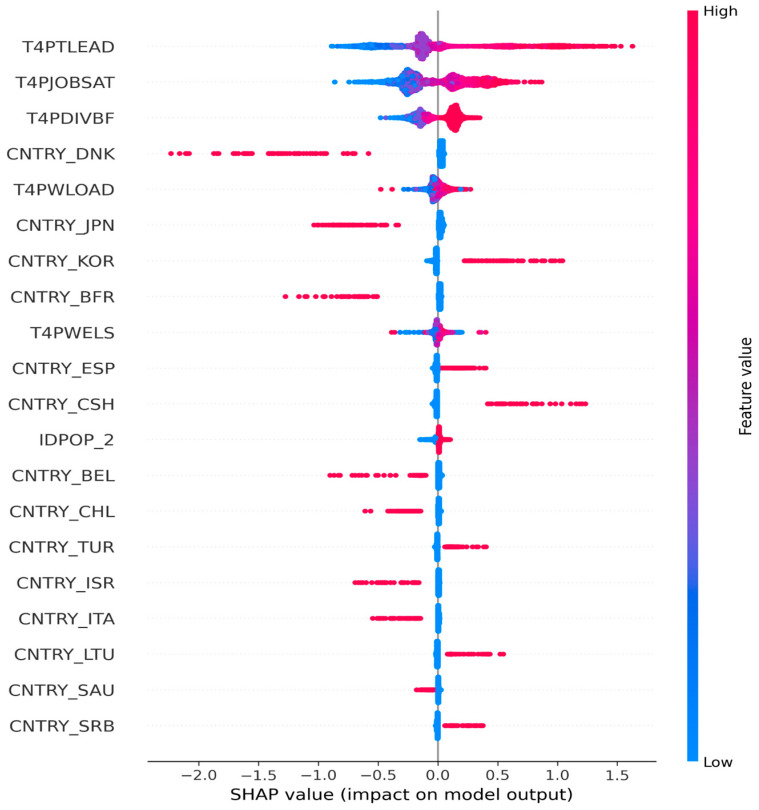
SHAP beeswarm graph.

The visualizations generated through the Random Forest model within the scope of SHAP analysis revealed a pattern largely consistent with the permutation importance findings. This indicates that the variables that stand out in predicting the participant school climate can be similarly determined using different explanatory techniques. Figure 6 shows the average absolute SHAP values of the variables, and Figure 7 shows the directional effects of the variables on the model output. Thus, it can be said that the machine learning findings are not only based on model performance but are also evaluated within an interpretable framework that can explain how each variable contributes to the model output. In this respect, SHAP analysis serves as a complementary tool that strengthens the research findings and reveals the role of the variables within the model in more detail.

Figure 6 shows the variables with the highest average absolute impact on the model output through a bar graph based on SHAP analysis. Since the average absolute SHAP value reflects the magnitude of a variable’s contribution to the model prediction, this figure reveals which variables are more decisive at the global level in predicting participatory school climate. The main message conveyed by the figure is that the model’s prediction process is not handled equally by all variables, but rather concentrated around certain variables. The findings show that school administrator leadership has by far the highest contribution value, followed by job satisfaction and diversity beliefs. This suggests that leadership behaviors, administrator job satisfaction, and inclusive school concepts play a central role in explaining participatory school climate. In contrast, while workload, well-being, and some country variables also contribute to the model prediction, their contribution is more limited compared to the top-ranking variables. Therefore, Figure 6 shows which variables the model explains most in the participatory school climate, demonstrating that the findings rely not only on predictive power but also on interpretability. Figure 7 shows the direction and magnitude of each variable’s effect on the model prediction at the observational level through a SHAP beeswarm graph. Unlike the bar graph in Figure 6, this graph reveals not only which variable is significant but also how low or high values of that variable affect the model output. In the graph, the horizontal axis shows the SHAP value, i.e., the variable’s effect on increasing or decreasing the prediction; the vertical axis lists the variables in order of importance. The color of the points reflects the level of the variable value; blue tones indicate low values, and pink tones indicate high values. In this respect, the figure explains how the model predicts the participatory school climate in a more detailed and interpretable way.

Examining Figure 7, it is seen that high values in the school administrator leadership variable are mostly associated with positive SHAP values, while low values are associated with negative SHAP values. This finding shows that as the leadership level increases, the model produces higher predictions regarding the participatory school climate. A similar pattern is observed for school administrator job satisfaction. It is understood that when job satisfaction is high, the model output is positively affected, while when it is low, the prediction is downwardly pressured. Similarly, in the diversity beliefs variable, it is seen that higher values contribute more positively. This pattern shows that beliefs about leadership, job satisfaction, and inclusion are not only important variables in explaining the participatory school climate but also that the direction of their effects is largely positive. In contrast, it is seen that the direction of the effect differs depending on the context in some country variables. For example, in some country dummy variables, high values affect the model output positively, while in others, there is a negative contribution. This situation suggests that the participatory school climate is shaped not only by individual and organizational characteristics but also in relation to the national context. In variables such as workload and well-being, it is understood that the effects are clustered in a narrower range, and therefore, their contribution to the model prediction remains more limited. Overall, Figure 7 reveals that leadership, job satisfaction, and diversity beliefs are particularly decisive in predicting the participatory school climate and that high levels of these variables mostly affect the model output positively. Thus, the figure shows that SHAP analysis reveals not only the significance of the variables but also the directional effects of the variables on the forecast.

### 4.4. Multilevel Mediation Analysis

In the second phase of the research, school administrator leadership and job satisfaction, which stood out as variables in machine learning analyses, were tested through a multilevel mediation model. In this model, school administrator leadership was considered as the independent variable, job satisfaction as the mediating variable, and participatory school climate as the dependent variable. Additionally, workload, diversity beliefs, and sample population were included as control variables. Within this scope, first, the effect of leadership on job satisfaction and, then, the combined effects of leadership and job satisfaction on participatory school climate were examined.

Table 3 shows the coefficients for direct, indirect, and total effects obtained from the multilevel mediation analysis. Examining the findings, it is seen that the effect of school administrator leadership on job satisfaction is positive and significant (a = 0.2121). Similarly, the effect of job satisfaction on participatory school climate is also positive and significant (b = 0.1419). The fact that the direct effect of leadership on participatory school climate retains its significance even after the mediating variable is included in the model (c = 0.1998) indicates that job satisfaction plays a partial mediating role in this relationship. The estimated indirect coefficient was 0.0301, and approximately 13.1% of the total association was consistent with an indirect pathway through school administrator job satisfaction. The interpretation of the indirect association focused on the size of the indirect coefficient and the bootstrap confidence interval. This approach was preferred because bootstrap confidence intervals provide more informative evidence for the stability of indirect associations in large samples. The bootstrap confidence interval did not include zero, indicating that the indirect association was stable across resampled estimates.

Table 4 shows the coefficients for the principal variables in the mediating and outcome models. The mediating model findings reveal that school principal leadership positively predicts job satisfaction, while workload has a negative effect on job satisfaction. Diversity beliefs, however, are found to positively contribute to job satisfaction. In the outcome model, it is noteworthy that both the national level of leadership and the national average leadership level produce significant and positive effects on the participatory school climate. This finding indicates that the participatory school climate is related not only to individual leadership perceptions but also to the average leadership level shaped within the national context.

Furthermore, the inclusion of job satisfaction as a significant predictor in the outcome model supports the mediating finding. The significant effects of workload and diversity beliefs on the outcome variable suggest that the participatory school climate has a multidimensional and multi-level structure.

**Figure 8:**
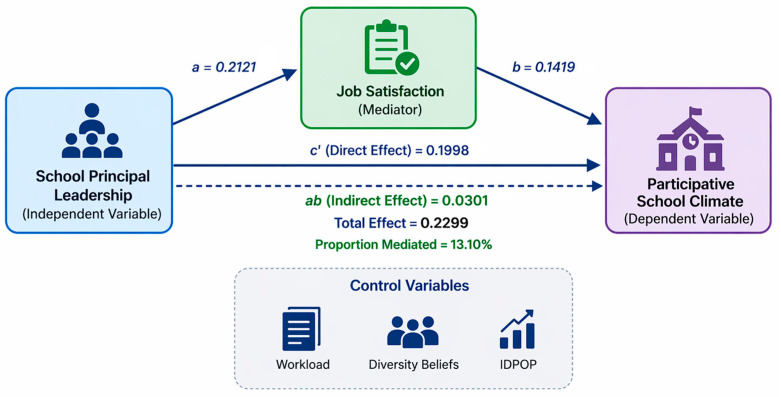
Multilevel mediation model. Note. IDPOP refers to sample population.

Figure 8 visually presents the direct and indirect pathways tested in the multi-level mediation model. In the figure, school principal leadership is shown as the independent variable, job satisfaction as the mediating variable, and participatory school climate as the dependent variable. It was also noted that workload, diversity beliefs, and IDPOP variables were controlled for. In this respect, the figure contributes to a clearer understanding of the structural relationships between leadership, job satisfaction, and participatory school climate by visualizing the coefficients presented in Table 3 and Table 4 within a more holistic framework.

Figure 8 shows that school administrator leadership has a positive and significant effect on job satisfaction, and job satisfaction positively predicts the participatory school climate. Furthermore, the direct effect of leadership on the participatory school climate remains significant. The presence of an indirect effect and the fact that a certain portion of the total effect operates through job satisfaction indicate that job satisfaction plays a partial mediating role in this relationship. In other words, school administrator leadership not only directly affects the participatory school climate but also contributes to its strengthening by increasing the administrator’s job satisfaction. This finding reveals that leadership processes and the administrator’s job satisfaction work together in the formation of a participatory school climate and that the relationship should be evaluated within a multi-level structure.

### 4.5. Bootstrap Verification

**Figure 9:**
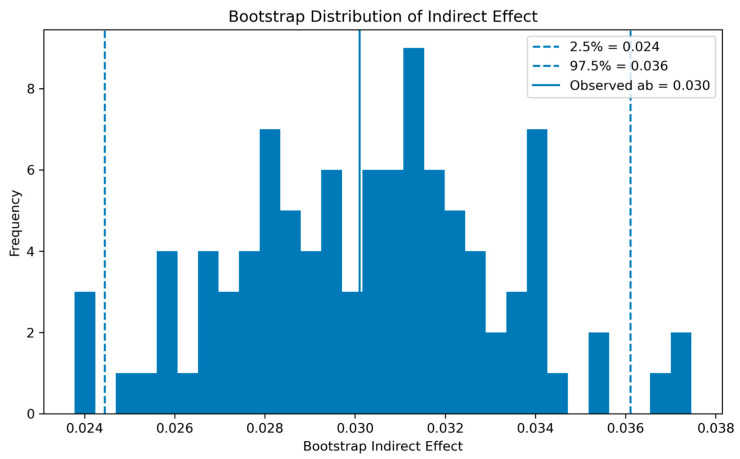
Bootstrap indirect effect distribution.

Bootstrap validation was performed to evaluate the robustness of the indirect effect obtained in the multilevel mediation analysis. The bootstrap approach is important because it shows the extent to which the indirect effect can be reliably reproduced across different resamples (Preacher & Hayes, 2008). In this context, the mean value of the indirect effect, the confidence interval, and the valid sample size were reported; the bootstrap distribution was also visually examined. Thus, it was evaluated not only whether the mediation finding was specific to the current sample but also to what extent it was statistically supported. Accordingly, Table 5 presents the summary statistics for the bootstrap indirect effect, and Figure 9 presents the distribution of the indirect effect coefficients.

Table 5 shows that the bootstrap mean indirect effect is 0.0303, and the 95% confidence interval ranges from 0.0245 to 0.0361. The absence of zero in the confidence interval indicates that the indirect effect is statistically significant and strongly supports the mediation finding. Furthermore, the inclusion of 100 valid resamples in the analysis suggests that the obtained result is not based on a random pattern and that the indirect effect is similarly maintained across different sample replications.

Figure 9 shows the distribution of the indirect effect coefficients obtained using the bootstrap method. In the histogram, each column reflects how frequently the indirect effect values calculated during the resampling process are generated within a specific range. One of the vertical lines in the graph shows the observed indirect effect value, and the other two show the lower and upper limits of the 95% confidence interval. This visual presentation allows for the evaluation not only of whether the indirect effect is significant but also to what extent it is consistently repeated across different resamples. Examining Figure 9, it can be seen that the bootstrap indirect effect values are largely clustered around 0.03. The concentration of the distribution around this center indicates that the indirect effect operating through job satisfaction is generated with similar magnitudes in different resamples. The fact that the observed indirect effect value is located quite close to the center of the distribution also reveals that the mediation coefficient obtained from the model is consistent with the bootstrap results. In other words, the indirect effect value obtained from the current sample is supported by the empirical distribution obtained through resampling. Another point that stands out in the graph is that the entire distribution is located in the region of positive values. Even the lower confidence interval remaining above zero indicates that the direction of the indirect effect is consistently positive. This suggests that the part of the effect of school principal leadership on participatory school climate that operates through job satisfaction is not due to random fluctuations but rather emerges in a stable positive direction. The relatively narrow confidence interval also indicates that the uncertainty level of the estimated indirect effect is limited and that the bootstrap results exhibit high stability. Therefore, Figure 9 visually demonstrates that the indirect effect obtained in the multi-level mediation analysis is not only statistically significant but also strong and reliable in terms of resampling-based validation. In this respect, the figure complements the bootstrap summary statistics presented in Table 5 and further supports the findings regarding the partial mediating role of job satisfaction in the relationship between leadership and participatory school climate.

## 5. Discussions

This paper is mainly focused on the two research questions that will be used to guide the research process. The first research question will focus on identifying the best predictors for participatory school climate using the school administrator data from the TALIS 2024 survey in an explanatory machine learning approach. The second research question will examine the mediating effect of job satisfaction between administrator leadership and participatory school climate.

In addition, this paper employs TALIS 2024 data from school administrators to examine participatory school climate using the proposed framework which combines the use of machine learning and multilevel mediation analysis. From the findings, it is clear that administrator leadership predicts participatory school climate in schools. Additionally, it was evident that job satisfaction acts as a predictor as well as part of the indirect path that connects leadership to participatory school climate. The increase in the performance of the models upon the inclusion of the countries’ information implies that it is necessary to consider contextual factors when analyzing participatory school climate, apart from individual and organizational variables. This finding is consistent with that of İncekara et al. (2026) based on their TALIS 2018 findings.

### 5.1. The Central Role of School Principal Leadership

The most consistent finding of the study is that principal leadership emerged as the strongest predictor of participatory school climate in both permutation importance and SHAP analyses. This result is in line with research showing that leadership is closely related to shared purpose, collaboration, trust, and the organizational functioning of schools. Leithwood et al.’s (2020) four-way model links school leadership to school outcomes through rational, emotional, organizational, and familial pathways, while Sebastian et al. (2017) show that principal and teacher leadership operate as interconnected processes within schools. In this respect, the present finding supports the view that participatory climate is closely associated with how school leaders encourage shared decision-making, collaboration, and mutual trust (Leithwood et al., 2020; Sebastian et al., 2017).

This interpretation is also consistent with recent studies connecting participation, collaboration, and innovation. Ham and Lee (2024) found that participatory decision-making is positively related to school innovation, particularly through schools’ capacity to cope with uncertainty. Similarly, Pan et al. (2024) showed that teacher collaboration is associated with school innovation by strengthening interaction patterns within the school. From this perspective, leadership appears important not simply because it creates formal decision-making structures but because it helps make collaboration, shared responsibility, and common goals part of everyday school practice (Ham & Lee, 2024; Pan et al., 2024).

### 5.2. The Function of Job Satisfaction as a Mediating Mechanism

The second important finding concerns the role of school administrator job satisfaction in the relationship between leadership and participatory school climate. The multilevel mediation results indicated that leadership was positively associated with job satisfaction and that job satisfaction was also associated with participatory school climate. The bootstrap results supported the stability of this indirect association. This suggests that participatory climate is related not only to leadership practices themselves but also to how school administrators experience their work, including their professional satisfaction, sense of meaning, and psychological resources.

This interpretation is consistent with research showing that school administrators’ working conditions are closely connected to both individual well-being and school functioning. Marsh et al. (2023) showed that school administrators’ mental health and well-being are shaped by the balance between job demands and work resources. Other studies similarly emphasize the relevance of work environment and leadership processes for educator satisfaction and school functioning (Eryilmaz et al., 2025; Sims, 2020). In this sense, job satisfaction should not be treated only as an outcome of leadership but also as part of the relational pathway through which leadership is associated with participatory school climate. For school improvement efforts, this means that administrators’ job satisfaction and well-being should be considered as part of the conditions that support a participatory school environment (Marsh et al., 2023).

### 5.3. Diversity Beliefs and the Inclusive Dimension of Participatory Climate

Diversity beliefs made a more limited but consistent contribution to the prediction of participatory school climate. This finding suggests that participatory climate is related not only to leadership and shared responsibility but also to openness to differences and inclusive school values. This interpretation is consistent with UNESCO’s 2024/5 Leadership in Education report, which defines educational leadership as a social function that brings people together around common goals and supports inclusive learning environments (UNESCO, 2024). It is also in line with studies showing that school climate is shaped not only by formal practices but also by shared cultural values, norms, and interaction patterns (Masry-Herzallah & Da’as, 2021; Hidayat & Patras, 2024). In this respect, the positive contribution of diversity beliefs suggests that participatory school climate should be understood through both procedural participation and cultural inclusion.

### 5.4. The Importance of Country Context and Multi-Level Patterns

One of the notable findings is that model performance improved when the country variable was included, and some country dummy variables ranked relatively high in importance. This suggests that participatory school climate is associated not only with leadership and job satisfaction but also with broader contextual conditions. The multilevel results support this interpretation by showing that both within-country leadership differences and country-level leadership averages were relevant to the model. Educational systems may differ in organizational norms, administrative cultures, accountability structures, and expectations about leadership. UNESCO’s 2024/5 report similarly emphasizes that educational leadership is shaped not only at the school level but also at local and national levels (UNESCO, 2024).

This interpretation is consistent with comparative education and school innovation research. Lu and Campbell (2021) argue that innovation and professional learning are embedded in educational contexts. TALIS-based findings by Pan et al. (2024) also show that school innovation should be considered together with collaboration and within-school interactions, while Ham and Lee (2024) suggest that the relationship between participatory decision-making and school innovation may vary according to cultural uncertainty avoidance. In this sense, the findings of the present study suggest that participatory school climate should not be interpreted through a single universal model. Future studies could examine these patterns through more detailed multilevel designs that include both school-level variables and system-level indicators (Lu & Campbell, 2021; Ham & Lee, 2024; Pan et al., 2024).

### 5.5. Methodological Contribution: Integrating Prediction and Explanation

In practical terms, machine learning techniques were used to predict the set of variables that would predict participatory school climate in the absence of any assumptions of linear relationships between variables. Prediction alone does not necessarily provide the reason or mechanisms for such relationships. For this reason, multilevel mediation analysis was used as the second step to test if there is an indirect effect of leadership on participatory school climate by way of job satisfaction, accounting for cross-national differences in such associations. The combination of these methods improves both practical significance and theoretical soundness: machine learning finds the important predictors, while mediation offers a possible explanation of the observed relationship.

A methodological contribution of this study is that it combines predictive machine learning with multilevel mediation analysis in the same research design. The machine learning models identified variables more strongly associated with participatory school climate, while SHAP and permutation importance analyses helped interpret their relative importance. The multilevel mediation analysis then examined whether job satisfaction, one of the prominent variables, was part of an indirect pathway between leadership and participatory school climate. In this way, the study brought together prediction and explanation without treating variable importance as causal evidence. This approach is consistent with recent work emphasizing the value of machine learning for capturing multivariate and nonlinear patterns in educational data (Masci et al., 2018), as well as with the explainable AI literature, which stresses the importance of interpretability in social science research (Lundberg & Lee, 2017; Molnar, 2020).

### 5.6. Practical Implications of the Findings

Although the XGBoost model produced the best prediction performance, the explained variance was modest (R^2^ = 0.218). This result should be interpreted with caution. However, modest levels of explained variance are common in educational and organizational research because school climate is shaped by many factors at the individual, school, and system levels. In this study, leadership, job satisfaction, diversity beliefs, workload, well-being, country, and sample population explained an important but limited part of the variation in participatory school climate. Therefore, the findings should not be read as a full explanation of participatory school climate. Rather, they show which variables were more informative within the available TALIS 2024 school administrator data. Future studies may improve explanatory power by including additional variables such as school resources, teacher collaboration, organizational trust, principal–teacher relationships, school size, community context, and local leadership structures. These variables may capture aspects of school climate that were not available in the current TALIS composite indicators.

When the findings are considered as a whole, it becomes clear that efforts to strengthen participatory school climate should not be limited to establishing formal decision-making mechanisms. To achieve more lasting and effective outcomes, it is necessary to address, in an integrated manner, professional development opportunities that support school principal leadership, working conditions that enhance principals’ job satisfaction and well-being, school-level structures that encourage collaboration, and school policies that make inclusive values visible. UNESCO (2024) emphasizes that the selection, preparation, and support of educational leaders are of strategic importance for education systems. Similarly, studies on school innovativeness and participatory decision-making show that processes such as trust, collaboration, and shared responsibility increase a school’s capacity for change (Ham & Lee, 2024; Pan et al., 2024). In this respect, the most important practical implication is that participatory school climate should not be treated merely as a “soft” variable under the heading of climate; rather, it should be regarded as a core area of school development that must be addressed together with leadership, well-being, inclusivity, and contextual support.

Specifically, professional development for school administrators must focus on processes that will allow the principal to develop the habit of shared decision-making, amplifying teacher voice, collaborative problem-solving, and building trust among members of the school faculty. Professional development for principals should not only involve information-gathering sessions but must include practice activities such as discussion of case studies, learning communities, mentorship, coaching, reflection on actual school challenges, and ongoing follow-up after training. All of these features are likely to help principals apply leadership skills learned through training to everyday activities at the school.

The results also imply that any attempts aimed at improving the participatory atmosphere at schools should take into account principals’ job satisfaction and well-being. In particular, they should reduce extraneous burdens associated with administrative tasks; provide for peer and supervisory support; create chances for professional acknowledgment; secure time for leadership in teaching and relationship-building; and provide well-being interventions that address issues of stress, role strain, and work–life conflict. In other words, promoting administrator well-being cannot be separated from improving conditions for school transformation since it is just one part of the overall process.

Based on these findings, several practical suggestions can be made. School leader training programs should include topics such as shared decision-making, teacher participation, collaborative problem-solving, and trust-building. These topics are important because leadership practices are closely related to school climate and school improvement (Hallinger, 2005; Leithwood & Jantzi, 2005; Spillane, 2006). Professional development for school administrators should also be connected to daily school practice. For this reason, coaching, mentoring, peer learning, and school-based activities may be useful. The findings also suggest that education systems should pay attention to school administrators’ job satisfaction and well-being. Job satisfaction is related to working conditions, workload, support, collaboration, and school climate (Bakker & Demerouti, 2017; Admiraal & Røberg, 2023; OECD, 2019, 2020). At the school level, participatory decision-making can be supported through teacher consultation, collaborative planning, and school climate teams. These practices may help schools develop a more shared and supportive climate. Finally, policies on participatory school climate should include inclusive leadership. This means respect for diversity, a sense of belonging, and cultural inclusion. Previous studies show that inclusive leadership is important for democratic and participatory school environments (Khalifa et al., 2016; Khalifa, 2020). Therefore, national and local education authorities should see participatory school climate as an important part of school improvement.

### 5.7. Limitations

Several limitations should be considered when interpreting the findings. First, the study used cross-sectional TALIS 2024 school administrator data. Although cross-sectional data are useful for examining relationships among variables, they do not allow the temporal ordering of these relationships to be tested directly (X. Wang & Cheng, 2020). Therefore, the associations among school administrator leadership, job satisfaction, and participatory school climate should not be interpreted as causal effects. This caution is particularly important for the mediation results because mediation analysis cannot provide strong causal evidence without temporal priority and adequate control of external confounders (S. E. Maxwell & Cole, 2007; S. E. Maxwell et al., 2011). Future studies using longitudinal, multi-wave, experimental, or quasi-experimental designs could test the direction and stability of these relationships more directly.

Second, the data were based on self-reports. TALIS data reflect the perceptions, beliefs, and statements of teachers and school administrators and may not fully correspond to administrative records, direct observations, or performance indicators. Social desirability, recall difficulties, and cultural response patterns may also introduce bias into self-report measures (Krumpal, 2013; OECD, 2025a; Podsakoff et al., 2003). Because several key variables were drawn from the same respondent source and survey context, common method bias cannot be ruled out. In addition, the analyses relied only on school administrator data and did not include teacher, student, or external stakeholder perspectives. Future studies using multi-source data could provide a more comprehensive assessment of participatory school climate.

Third, although the study used an international and multilevel data structure, contextual diversity could not be fully represented. TALIS 2024 analyses suggest using school weights for principal-only results and international model parameters to support cross-country comparability. However, these procedures do not fully capture system-level differences such as administrative culture, accountability regimes, school autonomy, funding structures, and professional norms (OECD, 2025a). In the present study, country context was addressed mainly through country variables and country-level clustering. Thus, while the findings indicate between-country differences, they cannot fully explain which specific policies, cultural conditions, or organizational arrangements are related to these differences. Future research should include system-level indicators, policy variables, and school-level structural characteristics to examine the contextual conditions associated with participatory school climate in more detail.

Fourth, the explainable machine learning approach also has limitations. Machine learning models are useful for identifying complex and nonlinear patterns, but variable importance rankings should not be interpreted as causal importance. Post hoc explainability methods such as SHAP show which patterns the model is sensitive to, but they may produce unstable or misleading weights when variables are highly correlated. These techniques make associations visible; they do not turn them into causal relationships (Huang & Marques-Silva, 2024; Lundberg et al., 2018; Gilpin et al., 2018). For this reason, the variable importance findings should be interpreted together with the theoretical framework and the multilevel modeling results. The modest model performance also suggests that participatory school climate is shaped by additional structural, cultural, and interpersonal factors not included in the present models.

Another limitation of this study is concerned with the list of covariates used in the analytical model. The study includes such covariates as workload, diversity attitudes, clustering by country, and sample as the independent variables but some other potentially significant factors have been omitted. For example, tenure, pay level, quality and type of professional education, ethnicity/race, and school budget can be related to both the job satisfaction of school administrators and school participation climate. Due to the lack of these variables in the analysis, the results of regression models can be affected by the omitted variable bias problem. This means that the reported effect can only be considered as an unadjusted association, not a cause-and-effect relation.

Finally, the variables included in the study consist of theoretically significant indicators that can be interpreted within the context of educational administration; however, this has somewhat limited the scope of the analysis. While participatory school climate appears to be closely related to variables such as leadership, job satisfaction, workload, and diversity beliefs, it can also be influenced by other organizational and contextual factors such as teacher collaboration, trust, school resources, professional learning communities, student composition, and local governance structures. Therefore, while the results of this study provide strong evidence regarding some key determinants of participatory school climate, they should not be considered a final and complete explanation encompassing all dimensions of this structure. In the future, how participatory school climate is formed and maintained can be investigated more deeply using broader sets of variables, multi-level longitudinal designs, and mixed methods approaches.

There are a number of other limitations that must be taken into account. The first one is that survey weights as well as the whole complex design of sampling was not accounted for in the analysis of the data, which means that the results show only those correlations that can be observed in the analytic sample but cannot be generalized to the whole population of interest for the respective education systems. The second limitation is that the variance explained by the best fitting model was rather modest (R^2^ = 0.218), meaning that there are some other variables that might influence school participation climate. For example, those can include local factors, the level of resources of a particular school, the experience of the administrator, the composition of teachers working in schools, the organizational history of schools, and so forth. Finally, the use of the data coming from self-reports of administrators increases the risk of method bias.

### 5.8. Future Studies

Several directions stand out for future research. First, studies using longitudinal data structures are needed to more clearly reveal the temporal aspect of the relationships identified in this research. This would allow for a more detailed examination of how the relationship between leadership, job satisfaction, and participatory school climate changes over time and under what conditions it strengthens or weakens. Furthermore, multi-source research that considers teacher, student, and school administrator data together can provide a comparative assessment of how participatory school climate is perceived by different stakeholders. In addition, more advanced multi-level models that consider system-level policy indicators, school characteristics, and cultural context variables together can more clearly reveal through which specific mechanisms the country context exerts its influence. Finally, qualitative and mixed-methods research can provide a deeper examination of how the relationship between leadership and participatory school climate is produced in the daily life of the school. In conclusion, this study reveals the central importance of school administrator leadership and job satisfaction in understanding participatory school climate; it also emphasizes the context-sensitive, relational, and multi-level nature of this structure. In this respect, the study contributes to the literature on educational administration while also offering a meaningful theoretical and practical framework for school improvement practices.

## 6. Conclusions

This study investigated the primary predictors of participatory school climate using TALIS 2024 school administrator data, employing a combination of explanatory machine learning and multilevel mediation analysis. The findings revealed that school administrator leadership is the strongest variable in explaining participatory school climate. School administrator job satisfaction was found not only to be a significant predictor but also to act as a partial mediator in the relationship between leadership and participatory school climate. Additionally, diversity beliefs were found to contribute positively, and the country context enhanced the explanatory power of the model. These findings suggest that participatory school climate should be considered not merely as an individual or managerial outcome, but as a multi-layered organizational structure shaped by leadership, job satisfaction, inclusivity, and contextual conditions. A significant contribution of this research is the integration of explanatory machine learning and multilevel modeling within the same analytical framework. The machine learning approach revealed variables more strongly associated with participatory school climate, along with their relative importance levels. Multilevel mediation analysis demonstrated the mechanisms by which these variables operate. Thus, the study not only identified which variables were stronger but also explained how this relationship was established. In this respect, the findings demonstrate that large-scale international datasets can be used in educational sciences not only for descriptive but also for explanatory purposes. From an application perspective, the findings indicate that efforts to strengthen a participatory school climate should not be limited to formal administrative arrangements. In this process, it is important to increase professional development opportunities that support school administrator leadership, create working conditions that will enhance the job satisfaction and well-being of administrators, strengthen intra-school processes that support collaboration and shared responsibility, and develop a school culture based on inclusive values. Therefore, a participatory school climate should be considered not only as a reflection of intra-school relations but also as a fundamental organizational area that should be at the center of school development policies.

## Figures and Tables

**Table 1:** Performance evaluation of ML approaches.

Dataset	Model	R^2^	MAE	RMSE
With country	XGBoost	0.218	1.417	1.794
With country	ElasticNet	0.210	1.438	1.803
With country	Random Forest	0.178	1.440	1.840
Without country	XGBoost	0.123	1.492	1.900
Without country	Random Forest	0.120	1.491	1.903
Without country	ElasticNet	0.102	1.518	1.923

**Table 2:** Prominent predictors according to permutation importance results.

Feature Number	With Country Model	Importance	Without Country Model	Importance
1	T4PTLEAD	0.096	T4PTLEAD	0.112
2	T4PJOBSAT	0.053	T4PJOBSAT	0.044
3	CNTRY_DNK	0.028	T4PDIVBF	0.018
4	CNTRY_JPN	0.020	T4PWELS	0.010
5	T4PDIVBF	0.016	T4PWLOAD	0.005
6	CNTRY_KOR	0.014	IDPOP_2	0.005

Note. T4PTLEAD = school administrator leadership; T4PJOBSAT = school administrator job satisfaction; T4PDIVBF = diversity beliefs; T4PWELS = school administrator well-being; T4PWLOAD = school administrator workload; CNTRY_DNK = Denmark; CNTRY_JPN = Japan; CNTRY_KOR = Korea; IDPOP_2 = sample population.

**Table 3:** Effect coefficients for the multilevel mediation analysis.

Effect	Coefficient
a path (Leadership → Job satisfaction)	0.2121
b path (Job satisfaction → Participatory school climate)	0.1419
Direct effect (c′)	0.1998
Indirect effect (ab)	0.0301
Total effect	0.2299
Mediation ratio	0.1310

**Table 4:** Key coefficients for the mediator and outcome models.

Model	Variable	B	SE	*p*
Mediator model	T4PTLEAD_within	0.2121	0.0074	<0.001
Mediator model	T4PWLOAD_within	−0.2619	0.0075	<0.001
Mediator model	T4PDIVBF_within	0.1354	0.0075	<0.001
Outcome model	T4PTLEAD_within	0.1998	0.0077	<0.001
Outcome model	T4PTLEAD_mean_ctry	0.5352	0.1606	<0.001
Outcome model	T4PJOBSAT_within	0.1419	0.0080	<0.001
Outcome model	T4PWLOAD_within	0.0596	0.0079	<0.001
Outcome model	T4PDIVBF_within	0.0945	0.0076	<0.001

Note. T4PTLEAD = school administrator leadership; T4PJOBSAT = school administrator job satisfaction; T4PWLOAD = school administrator workload; T4PDIVBF = diversity beliefs. The suffix “within” refers to within-country centered variables, and “mean_ctry” refers to the country-level mean.

**Table 5:** Summary of the bootstrap indirect effect.

Bootstrap Mean	Lower CI (2.5%)	Upper CI (97.5%)	Number of Valid Samples
0.0303	0.0245	0.0361	100

## Data Availability

The original data presented in the study are openly available in https://www.oecd.org/en/data/datasets/talis-2024-database.html, accessed on 5 November 2025.

## Appendix A

For readability, Section 4 presents construct labels in place of the original TALIS variable names. The mapping between these labels and the TALIS variable names is provided in Table A1.

**Table A1:** Mapping of TALIS Variable Names to Construct Labels Used in the Manuscript.

Raw Variables Names	Display Name in the Manuscript
T4PTLEAD	School administrator leadership
T4PJOBSAT	School administrator job satisfaction
T4PDIVBF	Diversity beliefs
T4PWLOAD	School administrator workload
T4PWELS	School administrator well-being
T4POPPART	Participatory school climate
IDPOP_2/IDPOP_3	Sample population
CNTRY_DNK	Denmark
CNTRY_JPN	Japan
CNTRY_KOR	Korea
CNTRY_ITA	Italy
CNTRY_BEL	Belgium
